# Bis[(*E*)-*N*-(pyridin-3-yl­methyl­idene)hydroxyl­amine-κ*N* ^1^]silver(I) perchlorate

**DOI:** 10.1107/S1600536812019290

**Published:** 2012-05-05

**Authors:** Jing Xu, Shan Gao, Seik Weng Ng, Edward R. T. Tiekink

**Affiliations:** a Key Laboratory of Functional Inorganic Material Chemistry, Ministry of Education, Heilongjiang University, Harbin 150080, People’s Republic of China; b Department of Chemistry, University of Malaya, 50603 Kuala Lumpur, Malaysia; c Chemistry Department, Faculty of Science, King Abdulaziz University, PO Box 80203 Jeddah, Saudi Arabia

## Abstract

Each of the ions in the title salt, [Ag(C_6_H_6_N_2_O)_2_]ClO_4_, is completed by the application of crystallographic twofold symmetry. The Ag^I^ atom is coordinated by two pyridine N atoms in an almost linear fashion [N—Ag—N = 170.0 (2)°], with the T-shaped coordination geometry being completed by a weakly associated perchlorate-O atom. Supra­molecular zigzag chains along [100] mediated by O—H⋯N hydrogen bonds [as parts of *R*_2_^2^(6) loops] feature in the crystal packing. The perchlorate O atoms are disordered over two sets of sites in a statistical ratio.

## Related literature

For structural diversity in the structures of silver salts, see: Kundu *et al.* (2010 ▶). For related structures, see: Abu-Youssef *et al.* (2010 ▶); Xu *et al.* (2012 ▶).
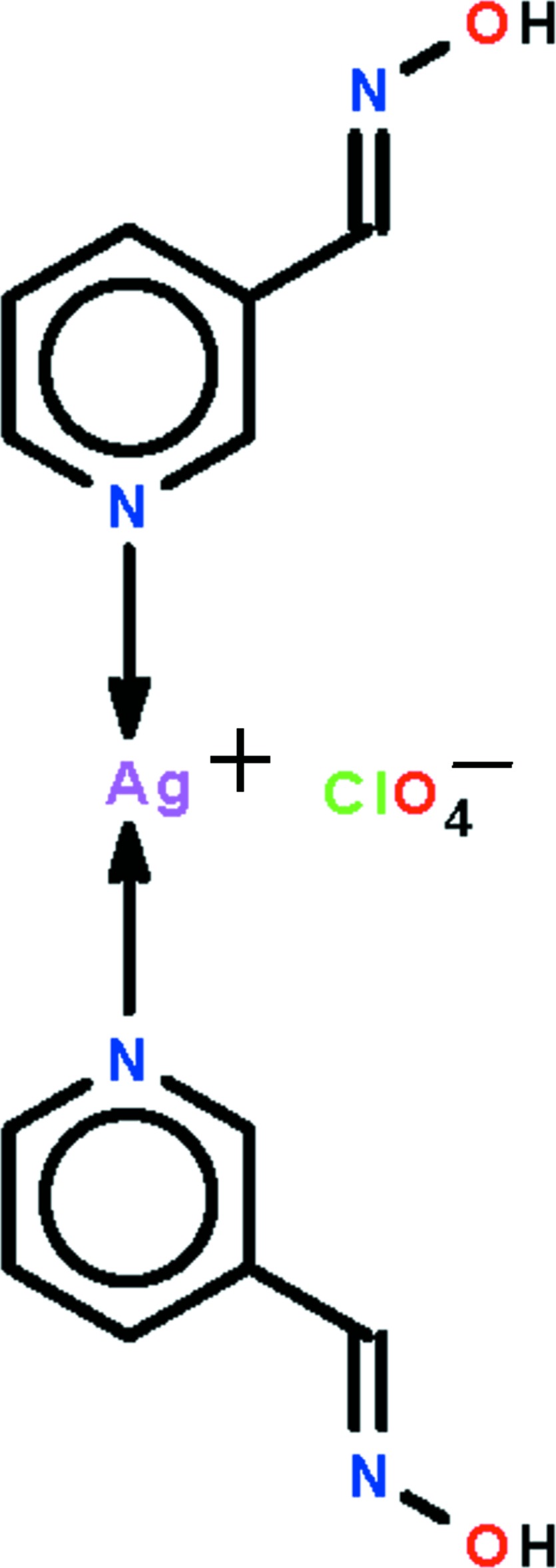


## Experimental

### 

#### Crystal data

[Ag(C_6_H_6_N_2_O)_2_]ClO_4_
*M* *_r_* = 451.58
Monoclinic, 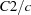

*a* = 15.382 (5) Å
*b* = 8.234 (3) Å
*c* = 13.320 (4) Å
β = 111.531 (15)°
*V* = 1569.3 (8) Å^3^
*Z* = 4
Mo *K*α radiation
μ = 1.49 mm^−1^
*T* = 293 K
0.18 × 0.16 × 0.14 mm

#### Data collection

Rigaku R-AXIS RAPID IP diffractometer
Absorption correction: multi-scan (*ABSCOR*; Higashi, 1995 ▶) *T*_min_ = 0.449, *T*_max_ = 1.000
7501 measured reflections
1795 independent reflections
1176 reflections with *I* > 2σ(*I*)
*R*_int_ = 0.052

#### Refinement

*R*[*F* ^2^ > 2σ(*F* ^2^)] = 0.048
*wR*(*F* ^2^) = 0.147
*S* = 1.06
1795 reflections
129 parameters
34 restraints
H-atom parameters constrained
Δρ_max_ = 0.74 e Å^−3^
Δρ_min_ = −0.49 e Å^−3^

### 

Data collection: *RAPID-AUTO* (Rigaku, 1998 ▶); cell refinement: *RAPID-AUTO*; data reduction: *CrystalClear* (Rigaku/MSC, 2002 ▶); program(s) used to solve structure: *SHELXS97* (Sheldrick, 2008 ▶); program(s) used to refine structure: *SHELXL97* (Sheldrick, 2008 ▶); molecular graphics: *X-SEED* (Barbour, 2001 ▶) and *DIAMOND* (Brandenburg, 2006 ▶); software used to prepare material for publication: *publCIF* (Westrip, 2010 ▶).

## Supplementary Material

Crystal structure: contains datablock(s) global, I. DOI: 10.1107/S1600536812019290/hb6761sup1.cif

Structure factors: contains datablock(s) I. DOI: 10.1107/S1600536812019290/hb6761Isup2.hkl

Additional supplementary materials:  crystallographic information; 3D view; checkCIF report

## Figures and Tables

**Table 1:** Selected bond lengths (Å)

Ag—N1	2.138 (5)
Ag—O2	2.843 (9)

**Table 2:** Hydrogen-bond geometry (Å, °)

*D*—H⋯*A*	*D*—H	H⋯*A*	*D*⋯*A*	*D*—H⋯*A*
O1—H1*o*⋯N2^i^	0.84	2.07	2.821 (7)	148

Symmetry code: (i) 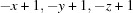.

**Fig. 1.**
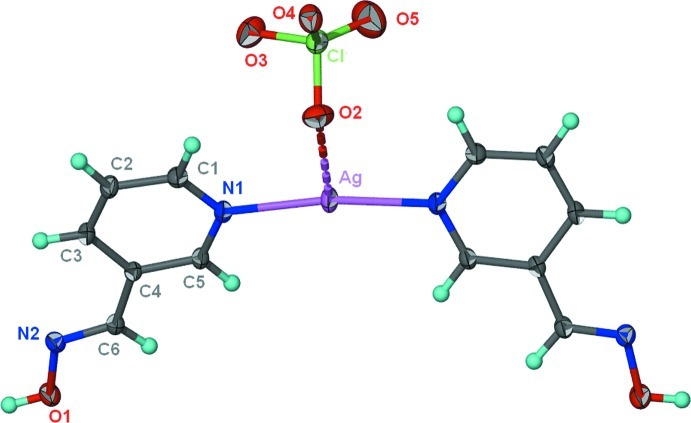
The molecular structure of (I) showing displacement ellipsoids at the 20% probability level. Each ion has crystallographic twofold symmetry. Only one orientation for the disordered perchlorate anion is shown.

**Fig. 2.**
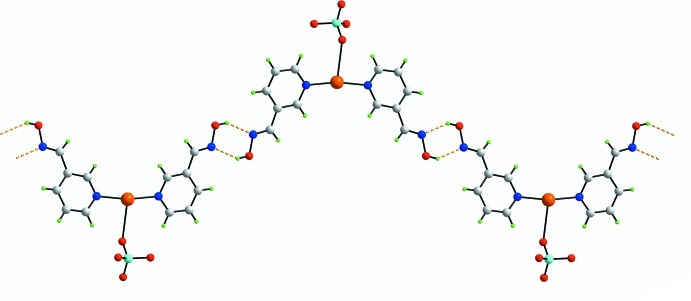
A view of the zigzag supramolecular chain along [100] in (I). The O—H···N hydrogen bonds are shown as orange dashed lines. Only one orientation for the disordered perchlorate anion is shown.

## supplementary crystallographic information

### Comment

The structural diversity of nitrogen adducts of silver salts are well known with distinct coordination geometries and supramolecular patterns being observed even when only a simple change, for example, in counter-ion, is made (Kundu *et al.*, 2010). The structural chemistry of pyridine-2-carboxaldoxime (LH) complexes with silver are relatively unexplored with only the Ag(LH)NO_3_ salt (Abu-Youssef *et al.*, 2010) and the salt co-crystal [C_12_H_11_AgN_4_O_2_]^+^[ClO_4_]^-^[C_12_H_12_AgN_4_O_2_] (Xu *et al.*, 2012) having been reported previously. Herein, the crystal structure determination of the title salt, [Ag(C_6_H_6_N_2_O)_2_]ClO_4_, (I), is described.

In (I), Fig. 1, each of the ions has crystallographic twofold symmetry. The Ag^+^ atom is coordinated by two N atoms in an almost linear array, Table 1. One of the perchlorate-O atoms is weakly associated with the silver atom so that the coordination geometry is T-shaped. The pyridine-2-carboxaldoxime ligand is planar as seen in the values of the C3—C4—C6—N2 and O1—N2—C6—C4 torsion angles of -2.3 (9) and -178.7 (5)°, respectively. The conformation about the imine bond [N2═C6 = 1.255 (7) Å] is *E*, and the nitrogen atoms are *anti*.

In the crystal packing, the oxime residues self-associate *via* O—H···N hydrogen bonds and six-membered {···HON}_2_ synthons, Table 2. The result is the formation of a supramolecular chain with a zigzag topology along the *a* axis, Fig. 2.

### Experimental

Silver perchlorate (1 mmol) and nicotinylaldehyde oxime (1 mmol) was dissolved in methanol solution (10 ml). The solution was filtered and set aside, away from light, for the growth of crystals. Colourless crystals deposited after several days.

### Refinement

Carbon- and oxygen-bound H-atoms were placed in calculated positions (C—H = 0.93 and O—H = 0.84 Å) and were included in the refinement in the riding model approximation, with *U*(H) set to 1.2–1.5*U*_eq_(C,*O*).

The oxygen atoms of the perchlorate ion are disordered about a twofold rotation axis, and four oxygen atoms were given 0.5 occupancies. The Cl—O distances were restrained to 1.41±0.01 Å and the O···O distances to 2.30±0.01 Å. The anisotropic displacement parameters of the oxygen atoms were restrained to be nearly isotropic.

### Crystal data

**Table:**

[Ag(C_6_H_6_N_2_O)_2_]ClO_4_	*F*(000) = 896
*M**_r_* = 451.58	*D*_x_ = 1.911 Mg m^−^^3^
Monoclinic, *C*2/*c*	Mo *K*α radiation, λ = 0.71073 Å
Hall symbol: -C 2yc	Cell parameters from 3613 reflections
*a* = 15.382 (5) Å	θ = 3.0–27.5°
*b* = 8.234 (3) Å	µ = 1.49 mm^−^^1^
*c* = 13.320 (4) Å	*T* = 293 K
β = 111.531 (15)°	Prism, colourless
*V* = 1569.3 (8) Å^3^	0.18 × 0.16 × 0.14 mm
*Z* = 4	

### Data collection

**Table:**

Rigaku R-AXIS RAPID IP diffractometer	1795 independent reflections
Radiation source: fine-focus sealed tube	1176 reflections with *I* > 2σ(*I*)
Graphite monochromator	*R*_int_ = 0.052
ω scan	θ_max_ = 27.5°, θ_min_ = 3.0°
Absorption correction: multi-scan (*ABSCOR*; Higashi, 1995)	*h* = −19→19
*T*_min_ = 0.449, *T*_max_ = 1.000	*k* = −10→10
7501 measured reflections	*l* = −17→17

### Refinement

**Table:**

Refinement on *F*^2^	Primary atom site location: structure-invariant direct methods
Least-squares matrix: full	Secondary atom site location: difference Fourier map
*R*[*F*^2^ > 2σ(*F*^2^)] = 0.048	Hydrogen site location: inferred from neighbouring sites
*wR*(*F*^2^) = 0.147	H-atom parameters constrained
*S* = 1.06	*w* = 1/[σ^2^(*F*_o_^2^) + (0.0776*P*)^2^ + 1.2356*P*] where *P* = (*F*_o_^2^ + 2*F*_c_^2^)/3
1795 reflections	(Δ/σ)_max_ = 0.001
129 parameters	Δρ_max_ = 0.74 e Å^−^^3^
34 restraints	Δρ_min_ = −0.49 e Å^−^^3^

### Fractional atomic coordinates and isotropic or equivalent isotropic displacement parameters (Å^2^)

**Table:**

	*x*	*y*	*z*	*U*_iso_*/*U*_eq_	Occ. (<1)
Ag	1.0000	0.04678 (8)	0.7500	0.0718 (3)	
Cl	1.0000	−0.4118 (3)	0.7500	0.0779 (6)	
O1	0.5863 (3)	0.6168 (5)	0.5648 (4)	0.0819 (13)	
H1o	0.5297	0.6370	0.5298	0.123*	
N1	0.8519 (3)	0.0695 (5)	0.6720 (4)	0.0573 (11)	
N2	0.6021 (3)	0.4515 (5)	0.5606 (4)	0.0603 (12)	
C1	0.7951 (4)	−0.0529 (6)	0.6259 (5)	0.0617 (14)	
H1	0.8207	−0.1559	0.6285	0.074*	
C2	0.7003 (4)	−0.0342 (7)	0.5744 (5)	0.0645 (14)	
H2	0.6630	−0.1234	0.5437	0.077*	
C3	0.6612 (4)	0.1150 (7)	0.5686 (4)	0.0587 (13)	
H3	0.5971	0.1289	0.5337	0.070*	
C4	0.7187 (3)	0.2477 (6)	0.6158 (4)	0.0495 (11)	
C5	0.8130 (3)	0.2174 (7)	0.6658 (4)	0.0544 (12)	
H5	0.8521	0.3044	0.6971	0.065*	
C6	0.6859 (4)	0.4136 (7)	0.6113 (4)	0.0571 (12)	
H6	0.7283	0.4943	0.6469	0.068*	
O2	1.0050 (8)	−0.2768 (10)	0.6778 (8)	0.105 (3)	0.50
O3	0.9040 (6)	−0.4057 (13)	0.7376 (10)	0.110 (4)	0.50
O4	1.0044 (7)	−0.5524 (9)	0.6836 (7)	0.089 (3)	0.50
O5	1.0649 (10)	−0.4065 (18)	0.8439 (7)	0.171 (6)	0.50

### Atomic displacement parameters (Å^2^)

**Table:**

	*U*^11^	*U*^22^	*U*^33^	*U*^12^	*U*^13^	*U*^23^
Ag	0.0392 (4)	0.0813 (5)	0.0859 (5)	0.000	0.0121 (3)	0.000
Cl	0.0624 (13)	0.0605 (12)	0.1049 (17)	0.000	0.0238 (13)	0.000
O1	0.064 (3)	0.054 (2)	0.106 (3)	0.005 (2)	0.005 (3)	0.007 (2)
N1	0.042 (2)	0.061 (3)	0.063 (3)	−0.0016 (19)	0.013 (2)	0.000 (2)
N2	0.054 (3)	0.054 (3)	0.066 (3)	0.005 (2)	0.013 (2)	0.006 (2)
C1	0.056 (3)	0.051 (3)	0.072 (3)	0.000 (2)	0.017 (3)	0.000 (3)
C2	0.048 (3)	0.058 (3)	0.075 (4)	−0.009 (3)	0.009 (3)	−0.006 (3)
C3	0.040 (3)	0.068 (3)	0.060 (3)	−0.003 (3)	0.009 (2)	0.002 (3)
C4	0.043 (2)	0.055 (3)	0.045 (2)	−0.006 (2)	0.010 (2)	0.002 (2)
C5	0.040 (3)	0.059 (3)	0.057 (3)	−0.005 (2)	0.010 (2)	−0.003 (2)
C6	0.045 (3)	0.057 (3)	0.062 (3)	−0.004 (2)	0.010 (2)	0.002 (2)
O2	0.123 (7)	0.082 (5)	0.125 (7)	−0.002 (5)	0.063 (6)	0.020 (5)
O3	0.098 (7)	0.104 (6)	0.148 (8)	−0.010 (6)	0.068 (7)	−0.018 (6)
O4	0.072 (5)	0.074 (5)	0.107 (6)	0.006 (4)	0.018 (5)	−0.014 (4)
O5	0.166 (11)	0.162 (9)	0.137 (9)	−0.001 (9)	−0.001 (8)	−0.008 (8)

### Geometric parameters (Å, º)

**Table:**

Ag—N1^i^	2.138 (5)	C2—H2	0.9300
Ag—N1	2.138 (5)	C3—C4	1.402 (8)
Ag—O2	2.843 (9)	C3—H3	0.9300
O1—N2	1.387 (7)	C4—C5	1.378 (7)
O1—H1o	0.8400	C4—C6	1.449 (8)
N1—C1	1.327 (7)	C5—H5	0.9300
N1—C5	1.346 (7)	C6—H6	0.9300
N2—C6	1.255 (7)	Cl—O5	1.283 (7)
C1—C2	1.374 (9)	Cl—O3	1.426 (7)
C1—H1	0.9300	Cl—O4	1.474 (6)
C2—C3	1.357 (8)	Cl—O2	1.490 (6)
			
N1^i^—Ag—N1	170.0 (2)	C5—C4—C3	117.1 (5)
N1^i^—Ag—O2	95.1 (3)	C5—C4—C6	118.6 (5)
N1—Ag—O2	94.2 (3)	C3—C4—C6	124.3 (5)
N2—O1—H1o	109.5	N1—C5—C4	124.0 (5)
C1—N1—C5	117.2 (5)	N1—C5—H5	118.0
C1—N1—Ag	124.1 (4)	C4—C5—H5	118.0
C5—N1—Ag	118.6 (3)	N2—C6—C4	122.0 (5)
C6—N2—O1	112.5 (5)	N2—C6—H6	119.0
N1—C1—C2	122.9 (5)	C4—C6—H6	119.0
N1—C1—H1	118.6	O5—Cl—O3	120.9 (7)
C2—C1—H1	118.6	O5—Cl—O4	114.9 (7)
C3—C2—C1	119.8 (5)	O3—Cl—O4	103.4 (5)
C3—C2—H2	120.1	O5—Cl—O2	113.4 (7)
C1—C2—H2	120.1	O3—Cl—O2	101.3 (5)
C2—C3—C4	119.1 (5)	O4—Cl—O2	100.0 (5)
C2—C3—H3	120.5	Cl—O2—Ag	117.8 (5)
C4—C3—H3	120.5		
			
N1^i^—Ag—N1—C1	168.7 (5)	Ag—N1—C5—C4	178.7 (4)
O2—Ag—N1—C1	9.2 (5)	C3—C4—C5—N1	−0.4 (8)
N1^i^—Ag—N1—C5	−9.2 (4)	C6—C4—C5—N1	−177.8 (5)
O2—Ag—N1—C5	−168.7 (4)	O1—N2—C6—C4	−178.7 (5)
C5—N1—C1—C2	−0.7 (9)	C5—C4—C6—N2	175.0 (5)
Ag—N1—C1—C2	−178.7 (5)	C3—C4—C6—N2	−2.3 (9)
N1—C1—C2—C3	0.5 (10)	O5—Cl—O2—Ag	−57.3 (9)
C1—C2—C3—C4	−0.2 (9)	O3—Cl—O2—Ag	73.9 (7)
C2—C3—C4—C5	0.1 (8)	O4—Cl—O2—Ag	179.9 (5)
C2—C3—C4—C6	177.4 (5)	N1—Ag—O2—Cl	−93.0 (6)
C1—N1—C5—C4	0.7 (8)		

Symmetry code: (i) −*x*+2, *y*, −*z*+3/2.

### Hydrogen-bond geometry (Å, º)

**Table:**

*D*—H···*A*	*D*—H	H···*A*	*D*···*A*	*D*—H···*A*
O1—H1*o*···N2^ii^	0.84	2.07	2.821 (7)	148

Symmetry code: (ii) −*x*+1, −*y*+1, −*z*+1.
